# Supplementary material for: TopoMetry systematically learns and evaluates the latent geometry of single-cell data
Source: eLife. 2026 Jul 3;13:RP100361. doi: 10.7554/eLife.100361 (PMC13331481; doi:10.7554/eLife.100361)
Supplement: Peer review history

# Editor report: eLife Assessment

**DOI:** 10.7554/eLife.100361.3.sa0

**Authors:** Jihwan Park

**Affiliations:** Gwangju Institute of Science and Technology Republic of Korea

**Keywords:** Convincing, Important

This **important** study presents a theoretically grounded framework for dimensionality reduction in single-cell RNA sequencing data, utilizing the principles of Riemannian manifolds. The proposed method addresses a critical challenge in bioinformatics-extracting highly informative latent dimensions without relying on the heuristic assumptions common in existing workflows. The evidence supporting the method's utility in estimating intrinsic dimensionality and identifying cell types is **convincing**, though the work would benefit from more rigorous validation against established ground truths and a clearer strategy for addressing prevalent batch effects.

# Referee report: Reviewer #1 (Public review)

**DOI:** 10.7554/eLife.100361.3.sa1

Summary:

Sidarta-Oliveira et al. present TopOMetry, a novel dimensionality reduction method based on the eigendecomposition of approximated Laplace-Beltrami Operator. Shortly, TopOMetry is an iterative version of the existing spectral methods (e.g., Laplacian Eigenmap or Diffusion map). It approximates the Laplacian operators twice, once in a "phenotypic space" and then once again in the eigenbases space. By doing this the approximated operator will contain more information of the manifold, which allows for more robust and accurate downstream analyses.

Strengths:

- Introduces operator-native fidelity scores and Riemannian diagnostics to single-cell analysis, enabling researchers to evaluate and trust embeddings - functionality absent in prior methods.

- The approach was rigorously tested based on synthetic and real single-cell RNA-seq datasets.

- The package is well-made and easily scalable to millions of cells.

- The comprehensive documentation helps the end-users to run desired analyses.

Weaknesses:

- The method is an extension of the current state-of-art methods, not a fundamentally new one.

Comments on revised version:

The revised manuscript partially addresses the concerns raised in the prior review. The jargon weakness has been substantially mitigated by relocating mathematical derivations to the Methods section and simplifying language in the main text; this weakness has been updated accordingly.

The introduction of operator-native fidelity scores and Riemannian diagnostics represents a meaningful addition and has been added to the Strengths. The benchmarking scope has also been notably expanded.

The core weakness - that the method is an extension of existing spectral methods rather than a fundamentally new contribution - remains unchanged, as the authors' rebuttal did not provide a sufficiently precise mathematical argument to overturn it.

# Referee report: Reviewer #2 (Public review)

**DOI:** 10.7554/eLife.100361.3.sa2

Summary:

This work introduces a novel framework to systematically learn the latent dimensions of single-cell data, grounded in the theory of the Riemannian manifold. The authors demonstrate how this framework can be applied to various important tasks, such as estimating intrinsic dimensionalities, annotating cell types, etc. They did a great job of tackling an important but not yet established problem in the field and approaching it with a theoretically sound and novel approach. I think after a more rigorous and comprehensive validation, this work could be impactful.

Strengths:

- Dimensionality reduction is a routine step in analyzing many high-dimensional data, such as molecular data. While the downstream analysis results depend heavily on this step, existing methods rely on strong assumptions and are sometimes heuristic. The authors present a novel, theoretically grounded approach to address this important problem.

- The authors demonstrated its usability in downstream analysis in a comprehensive manner. Especially, they show evidence suggesting novel T-cell subpopulations.

- I commend the authors for releasing and maintaining their software well with comprehensive documentation. This significantly increases the usability and accessibility of the method.

Weaknesses:

- The paper lacks experiments that validate the results. It would be beneficial to see additional evaluation settings with better-established ground truths to more strongly demonstrate the method's effectiveness.

- Batch effects are prevalent in single-cell data. The paper does not adequately address how the proposed method handles this issue.

# Author comment: Author response

**DOI:** 10.7554/eLife.100361.3.sa3

**Authors:** David S Oliveira, Ana I Domingos, Licio A Velloso

**Affiliations:** University of Oxford Oxford United Kingdom; University of Oxford Oxford United Kingdom; Universidade Estadual de Campinas (UNICAMP) CAMPINAS Brazil

The following is the authors’ response to the original reviews.

**Reviewer #1 (Public review):**

(1) The method is an extension of the current state-of-art methods, not a fundamentally new one.

We respectfully disagree with this characterization. While TopoMetry is inspired by the theory of spectral geometry, it is not a simple extension of existing dimensionality reduction methods such as Diffusion Maps. Instead, TopoMetry introduces a new framework for single-cell analysis that:

Iteratively approximates manifold geometry by constructing refined diffusion operators on spectral scaffolds (“the geometry of the geometry”), a procedure not present in existing methods.

Provides a unified workflow for dimensionality estimation, clustering, visualization, imputation, lineage inference, and diagnostics, all within the same geometric framework.

Introduces operator-native fidelity scores and Riemannian diagnostics to single-cell analysis, enabling researchers to evaluate and trust embeddings—functionality absent in prior methods.

Thus, TopoMetry represents a new paradigm for geometry-aware single-cell analysis, not merely a reimplementation of existing algorithms.

(2) The paper contains a lot of jargon.

We have thoroughly simplified the text throughout the manuscript. We now introduce geometric concepts in accessible terms, avoiding technical details where they are not essential for biological interpretation. For example, references to the Laplace–Beltrami operator and its eigenfunctions have been reduced and reframed in terms of “geometry,” “diffusion,” and “spectral scaffolds,” which are more intuitive for a general audience.

**Reviewer #1 (Recommendations for the authors):**

(1) What happens if the LBO is approximated more than twice? As the main idea of the method is an iterative approach to approximate LBO more precisely, then the authors would have already considered this. If so, this could be additionally discussed in the manuscript.

We thank the reviewer for this important point. Indeed, TopoMetry’s design naturally supports iterating the Laplace–Beltrami operator (LBO) approximation beyond two steps. However, additional iterations (three or more) lead to only marginal improvements in final results while significantly increasing computational cost. In some tested cases, additional iterations could even over-smooth the data, reducing the resolution of fine-scale structure. The revised manuscript avoids an excessive focus on iterative LBO approximations and instead centers the narrative around representing and evaluating the underlying geometry of single-cell data.

(2) As the paper describes the method in a very comprehensive way, as a result, it contains a lot of mathematical equations and jargon. This could hinder the visibility of the whole manuscript to biologists who do not have a background in mathematics. Thus, I strongly recommend that the authors consider moving a considerable amount of text to the supplementary material, and the main text should focus on the benchmarking results and the possible applications.

We appreciate this recommendation and have substantially revised the manuscript to make it more accessible to a broad biological audience. In the revised version:

We moved detailed mathematical derivations and operator definitions to the Methods section, keeping only the most essential concepts in the main text.

We reframed technical terms (e.g., Laplace–Beltrami operator, eigenfunctions) in simpler and more intuitive language in the main text.

The Results section now emphasizes benchmarking outcomes and biological applications.

**Reviewer #2 (Public review):**

(1) To encourage the single-cell community to adopt this method, the authors should more clearly demonstrate its advantages over existing methods. There are many single cell analysis algorithms that are proposed in each task and some of them are widely used by biologists. However, the comparison in this work is somewhat limited. For example, Even methods mentioned in the relevant work paragraph (2nd paragraph) on page 2 are not all compared, or the reason why they are not included is not discussed. Also, I am curious how PC dimensions are determined. The choice of 300 PCs on page 11 seems arbitrary. Furthermore, the usefulness of dimension-reduced data also depends a lot on the preceding processing steps, such as highly variable gene selection. I understand it is hard to control all those factors, but I think there is room for improvement.

We have substantially expanded the benchmarking and discussion of competing methods. These additions more clearly demonstrate TopoMetry’s advantages and robustness compared to widely adopted alternatives. In the revised manuscript:

We now benchmark TopoMetry against 68 diverse single-cell datasets, far exceeding the scope of the original version.

We explicitly compare TopoMetry with PCA→UMAP, standalone UMAP, and scVI. These workflows represent the *de facto* current standard in single-cell analysis. While numerous other approaches exist, a comprehensive benchmark of every possible workflow lies beyond the scope of this study and would itself warrant a dedicated report.

We adopt the exact same preprocessing steps for all evaluated workflows to ensure a fair comparison, except for scVI, which requires gene counts data and performs its own internal preprocessing.

We adjust the number of PCs used for each dataset based on the currently adopted “elbow point” *ad hoc*.

(2) The paper lacks experiments that validate the results. It would be beneficial to see additional evaluation settings with better-established ground truths to more strongly demonstrate the method's effectiveness.

We agree that validation is crucial and have strengthened this aspect:

We introduce new geometry-preservation metrics and validate that TopoMetry outperforms current *de facto* standards.

We demonstrate that TopoMetry resolves well-established ground-truth structures, such as the cell cycle in pancreas development and T cell proliferation, which PCA→UMAP fails to capture (Suppl. Fig. S3).

We validate the biological relevance of novel T cell subpopulations by linking them to TCR clonotypes and clonal expansion patterns using datasets with paired VDJ information (ECCITE-TCR, TICA).

We show that TopoMetry faithfully recovers expected lineage trajectories in atlas-scale datasets (MOCA).

These analyses demonstrate that TopoMetry not only preserves geometry but also recovers biologically meaningful ground-truth structures. Further experimental investigation of biological insights obtained from the presented examples exceeds the scope of the presented methodological work.

(3) The effect of various parameters, such as those involved in k-nearest neighbors (KNN) or choosing the appropriate Laplacian operator, is not comprehensively explored. How can we ensure the analysis is not overly sensitive to these parameters?

We now explicitly address parameter robustness and show that results are stable across a wide range of k values (30–200) in the neighborhood graph (Suppl. Fig. S1e).

The range of possible Laplacian operators was a design choice aimed at increasing user freedom, but we agree with the reviewer that this option could confuse readers and users. TopoMetry now only uses the appropriate operator (density-normalized graph Laplacian, a.k.a. diffusion operator), reducing variability and improving usability.

(4) Batch effects are prevalent in single-cell data. The paper does not adequately address this issue.

Several of the datasets we analyzed include cells from multiple donors and experimental batches, and TopoMetry successfully recovers consistent biological structure across these.

TopoMetry’s spectral scaffolds can be integrated with data integration methods such as Harmony and Scanorama, which are employed to correct the latent PCA space in current practice.

**Reviewer #2 (Recommendations for the authors):**

(1) The paper introduces technical jargon without sufficient explanation abruptly many times. This makes it difficult for readers from a biological background to follow. Even I, with a more computational background, struggled to grasp some parts.

We thank the reviewer for this feedback and have streamlined terminology throughout the manuscript, replacing jargon with more intuitive language and providing brief explanations when technical terms are first introduced. This makes the text more accessible to both computational and biological audiences.

(2) There is no comparison of the computational cost of this method with existing approaches, which is an important factor for practical adoption. Including a benchmarking section on this would be useful.

We thank the reviewer for this suggestion and have now included a runtime benchmark against PCA→UMAP, PHATE, and scVI (Suppl. Fig. 1f), showing that while TopoMetry is slightly slower than PCA→UMAP, it scales more favorably than alternative geometry-aware methods (PHATE) and neural networks (scVI).

(3) TopOMetry allows users to obtain and evaluate dozens of possible representations. However, I wonder if this could introduce a user burden, increasing uncertainty and subjectivity, as users should examine them manually. I think this should be clarified.

We appreciate this concern and have streamlined the workflow to minimize user burden. As shown in the original manuscript, representations learned with different TopoMetry kernels and Laplacian variants converge to highly similar results. Based on this, TopoMetry now defaults to the best-performing kernel and the most appropriate Laplacian operator, yielding only two scaffold representations (fixed-time and multiscale) and corresponding visualizations rather than dozens of alternatives. This removes the need for manual selection while retaining flexibility for advanced users. In addition, we introduced a single-line command that runs the entire analysis and generates a comprehensive PDF report, allowing users to evaluate results in a standardized and user-friendly way. Together, these changes eliminate unnecessary subjectivity and ensure consistent outputs across analyses.

(4) Formatting. There are errors in figure numbering within the main text. For instance, it should be Figure 4 instead of Figure 3 on page 11. Some figures are not concise. For example, Figure 2 contains too much text, which detracts from its visibility. I recommend trimming the figures to improve clarity. A color map is missing in Figure 2, which could help better interpret the data.

We have thoroughly adjusted the manuscript and figures for improved visibility and clarity.

Broader Impact and Reception

Since our preprint, TopoMetry has been used by Hale et al. (Science, 2024), where it helped reveal morphological T cell subpopulations, and in a recent preprint by Tedeschi et al. (2025). These independent applications highlight the utility and impact of TopoMetry beyond our group, supporting its relevance to diverse biological contexts. In addition, two independent studies performing multimodal integration of RNA and TCR data (Zhang et al., 2023 and Drost et al., 2024) have identified a diversity of T cell subpopulations that resembles the clusters identified by TopoMetry using only RNA data.
